# Supplementary material for: Bright, high-repetition-rate water window soft X-ray source enabled by nonlinear pulse self-compression in an antiresonant hollow-core fibre
Source: Light Sci Appl. 2021 Feb 12;10:36. doi: 10.1038/s41377-021-00477-x (PMC7881106; doi:10.1038/s41377-021-00477-x)
Supplement: Supplementary file 1 — Supplementary information. [file 41377_2021_477_MOESM1_ESM.docx]

Supplementary Information for

Bright, high-repetition-rate water window soft X-ray source enabled by nonlinear pulse self-compression in an antiresonant hollow-core fibre

M. Gebhardt,^1,2*^ T. Heuermann,^1,2^ R. Klas,^1,2^ C. Liu,^1,2^ A. Kirsche,^1,2^ M. Lenski,^1^ Z. Wang,^1^ C. Gaida,^1+^ J. E. Antonio-Lopez,^3^ A. Schülzgen,^3^ R. Amezcua-Correa,^3^ J. Rothhardt,^1,2,4^ and J. Limpert,^1,2,4^

^1^Institute of Applied Physics, Abbe Center of Photonics, Friedrich-Schiller-Universität Jena, Albert-Einstein-Str. 15, 07745 Jena, Germany

^2^Helmholtz-Institute Jena, Fröbelstieg 3, 07743 Jena, Germany

^3^CREOL, College of Optics and Photonics, University of Central Florida, Orlando, Florida 32816, USA

^4^Fraunhofer Institute for Applied Optics and Precision Engineering, Albert-Einstein-Str. 7, 07745 Jena, Germany

^+^now with Active Fiber Systems GmbH, Ernst-Ruska-Ring 17, 07745 Jena, Germany

*Corresponding author: [martin.gebhardt@uni-jena.de](mailto:martin.gebhardt@uni-jena.de)

This supplementary information contains Fig. S1-S7 and the table S1. It compares this work to state-of-the-art HHG in the water-window and gives a detailed characterization of the nonlinear pulse compression input and output. Subsequently, detailed numerical calculations of the pulse evolution in the ARHCF are presented and compared to the measurements. Note that the numerical treatment of the HHG is using solely on-axis quantities and represents a simplified, yet well-fitting description. At the end of this supplementary information, we present the results of a near-edge X-ray absorption fine structure (NEXAFS) spectroscopy measurement near the carbon K-edge as well as a long-term characterization of the power in the water-window in order to show the applicability of the source described herein.

The reference list used in the main manuscript is continued in this supplementary information.

**Overview of laser-driven HHG sources delivering high photon flux at the carbon K-edge**

Fig. S1 provides an overview of the approximate flux delivered from laser-driven HHG sources in a spectral region just above the carbon K-edge (300 eV). We note that the flux values stated in most literature (or calculated from the available information), as well as the result demonstrated in this work, are estimate values. The graph should draw the reader’s attention to the fact that this work achieves a significant, application-relevant flux at the carbon K-edge with almost two orders of magnitude lower peak power as compared to state-of-the-art water window HHG. At the same time, it represents the highest photon flux and efficiency demonstrated to date using >1kHz repetition rate.


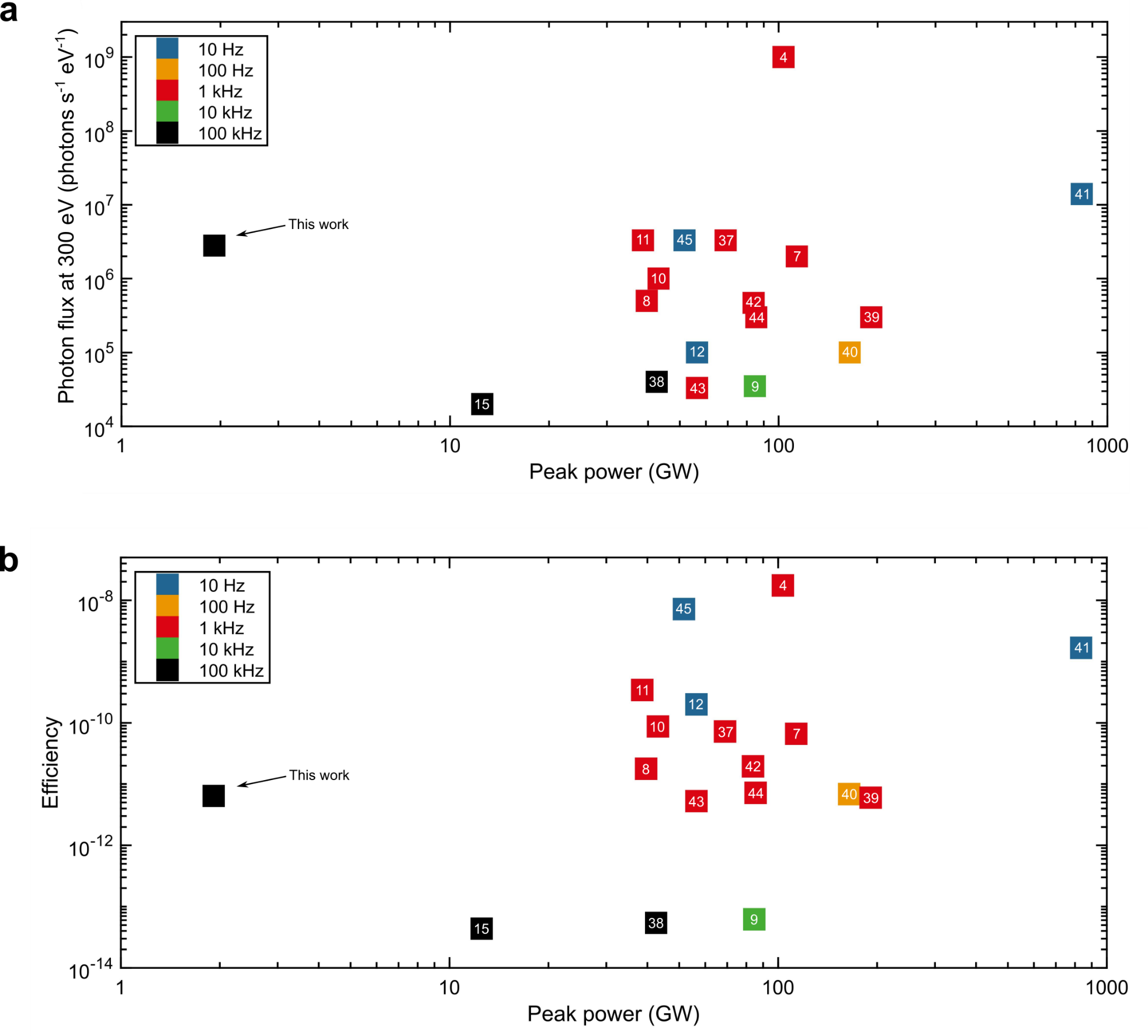


**Fig. S1 Laser-driven HHG sources at the carbon K-edge**^4,7–12,15,37–45^**.** **a,** Photon flux at 300 eV within a bandwidth of 1 eV, delivered by laser-driven HHG sources vs. the driving pulse peak power. **b,** Conversion efficiency, w.r.t. a bandwidth of 1 eV around 300 eV. Different colours represent different repetition rates and show that high repetition rate lasers were not able to produce >4×10^4^photons s^-1^ eV^-1^ at the carbon K-edge prior to this work.

**Ultrafast thulium-doped fibre laser performance**

The system layout of the ultrafast thulium-doped fibre chirped-pulse amplification system is described in the methods section. Fig. S2 gives an overview of the system performance at the optimized high-order harmonic generation experimental conditions. At this point, the system delivers 28.5 W of average power, which corresponds to 290 µJ of pulse energy at a 98 kHz repetition rate. We note that this is about 65% of its design performance level. The spectrum of the pulse train is depicted in Fig. S2a. The apparent modulations are due to residual water vapor absorption in the low-power section of the laser and the onset of nonlinear phase accumulation during amplification in the main amplifier. A collimated beam profile can be seen in the inset of Fig. S2a, showing the hexagonal active core structure of the used thulium-doped large-pitch fibre^46^. Fig. S2b shows the measured intensity autocorrelation (AC), from which a pulse duration of 103 fs (FWHM) is retrieved. This value is only 8% higher than the spectrum’s transform limit. The autocorrelation deconvolution factor is retrieved from the numerical data depicted by the red line in Fig. S2b. We generate the numerical description of the temporal pulse profile based on the measured laser spectrum. We add the spectral phases corresponding to the nonlinear propagation in the main amplifier as well as to a slight detuning of the stretcher/compressor in the chirped-pulse amplification system’s dispersion management. These phases result in a minor lengthening of the short main feature (shaded area in the inset of figure S2b) and a redistribution of less than 20% of the total pulse energy to satellite pulses. The retrieved pulse peak power is about 2 GW at this point. As can be seen from the comparison with the autocorrelation measurement, the agreement between the numerical and the experimental data is satisfying. Therefore, the description of the temporal pulse shape is used as the input for pulse propagation simulations, that result in the temporal on-axis intensity evolution shown in Fig. 2b of the main text.


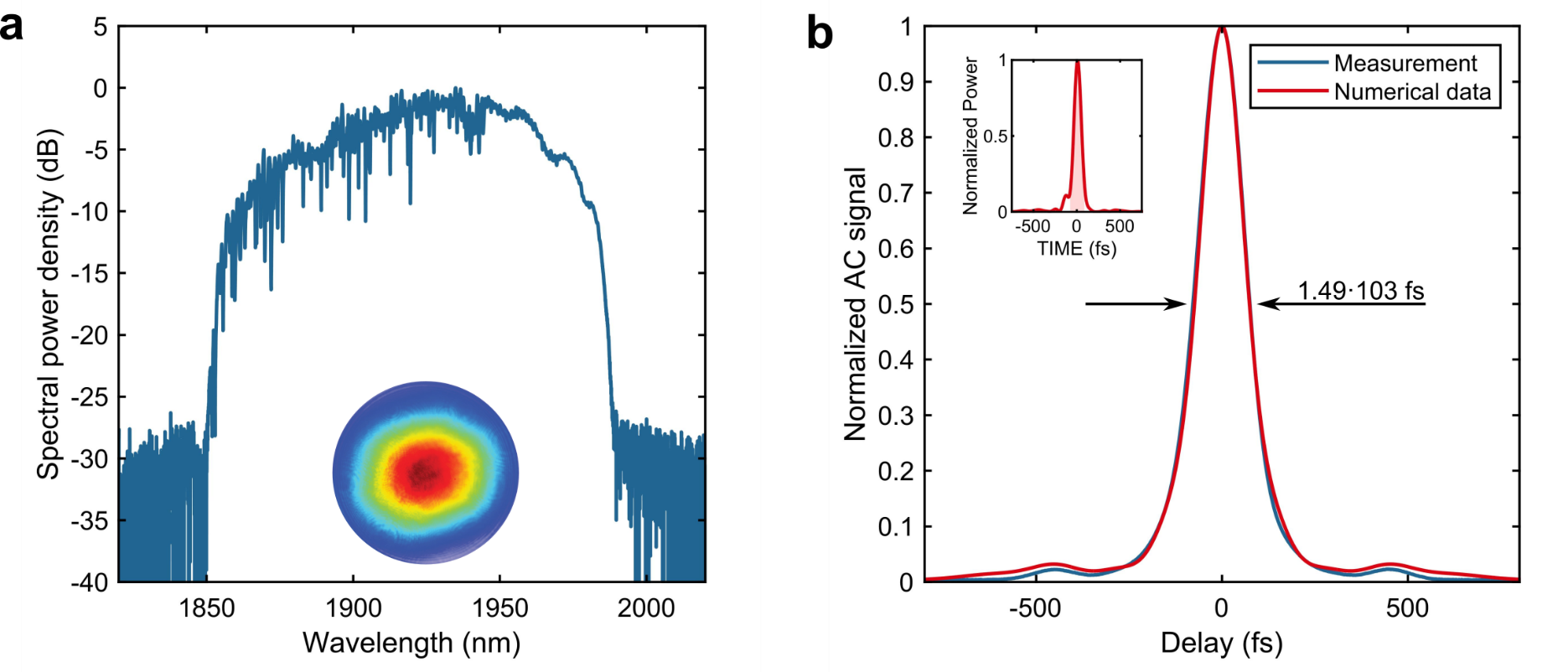


**Fig. S2 Characterization of the ultrafast thulium-doped fibre laser performance. a,** Spectrum of the laser output when the system performance was set to the experimental conditions for optimum HHG. Inset: image of the collimated beam profile. **b,** Normalized, measured autocorrelation signal (blue line) compared to the AC of the numerical data describing the temporal pulse shape (red line). Inset: Normalized temporal power envelope of the retrieved pulse shape, the shaded area contains ~80% of the total pulse energy.

**Experimental characterization and numerical simulation of the nonlinear pulse self-compression**

The antiresonant hollow-core fibre is drawn in-house at the University of Central Florida. A cross-section of the fibre used for the experiments is shown in Fig. S3a. The picture is recorded with the help of a calibrated microscope. The fibre core diameter is 84.5 µm, which is determined by fitting a circle with the corresponding dimensions (top left inset in Fig. S3a) into the centre of the 7-capillary structure. The bottom left inset shows an image of the output beam profile that is, with respect to the real fibre output facet dimensions, recorded with a magnification of a factor of 20. As compared to the scale of the fibre cross-section in Fig. S3a, the image is magnified by a factor of 1.7 for convenient display. From this measurement, we infer a mode-field diameter of 61.4 µm, which we use for the numerical description of the pulse evolution along the fibre. When excited in its fundamental mode, the overall transmission of the 120 cm long ARHCF is as high as 90%, including coupling losses.

Fig. S3b presents a comparison of the experimentally recorded and the simulated output spectra for the conditions of optimum HHG signal at 300 eV. These conditions are summarized in the table below.

**Table. S1 Summary of experimental conditions and simulation parameters for the nonlinear self-compression and simultaneous HHG.**

|  | Experiment | Simulation |
| --- | --- | --- |
| Fibre length | 120 cm | |
| Mode-field diameter | 61.4 µm | |
| Pressure at fibre input | 1.0 bar | |
| Pressure at fibre output | 3.8 bar | |
| Laser pulse energy | 290 µJ |  |
| Launched pulse energy |  | 248 µJ |

The measurement of the compressed pulse spectrum during the HHG experiments is possible due to a reflection from the annular mirror in the separation section of the soft X-ray beamline. As can be expected, both spectra shown in Fig. S3b feature significant broadening of the initial bandwidth, blueshift of the centre of mass due to ionization, and they are limited by a resonance band of the fibre at wavelengths <1380 nm.

The characterization of the self-compressed pulses is performed with a dispersion minimized interferometric autocorrelator and a pair of custom chirped mirrors, that compensate the dispersion of a 1 mm thick fused silica window, through which the laser is sent out of the sealed beamline. Fig. S3c presents a comparison of the measured and simulated autocorrelation traces. While there is a slight dephasing of the interference fringes in the wings of the autocorrelation, both traces show good agreement. It can be concluded that the simulated input and output characteristics are in good agreement with our measurements in both the temporal as well as the spectral domain. Therefore, it is inferred that the pulse evolution in the fibre is described to a reasonably accurate level by the numerical modelling. That evolution, in the form of the time-dependent on-axis intensity, is shown in Fig. S3d. Its obvious similarity to the simulation results presented in Fig. 2b of the main text shows that: (i) Our assumptions for the driving field that feeds in the expectations for the HHG phase matching are very close to the experimental reality. (ii) The pulse self-compression can be recovered in its major characteristics when energy and output pressure are slightly tuned (see main text).

Fig. S3e shows the soliton number during the temporal compression, retrieved from the numerical data. The soliton number *N* calculates as^47^:

$N= \sqrt{\frac{\gamma\hat{P}t_{\mathrm{FWHM}}^{2}}{3.11\cdot\left| \beta_{2} \right|}}$

where $\gamma, \hat{P}, t_{\mathrm{FWHM}}, \beta_{2}$ are the nonlinear parameter, the pulse peak power, its FWHM duration and the group velocity dispersion (also including the current central wavelength and plasma density), respectively.

While *N* is first increased due to the increasing nonlinearity associated with the pressure gradient, it falls towards unity as the pulses propagate to the fibre end. This behaviour is well-known from soliton pulse compression and it has been reported that self-compressed, blue-shifting solitons oscillate around the order $N=1$^48^.


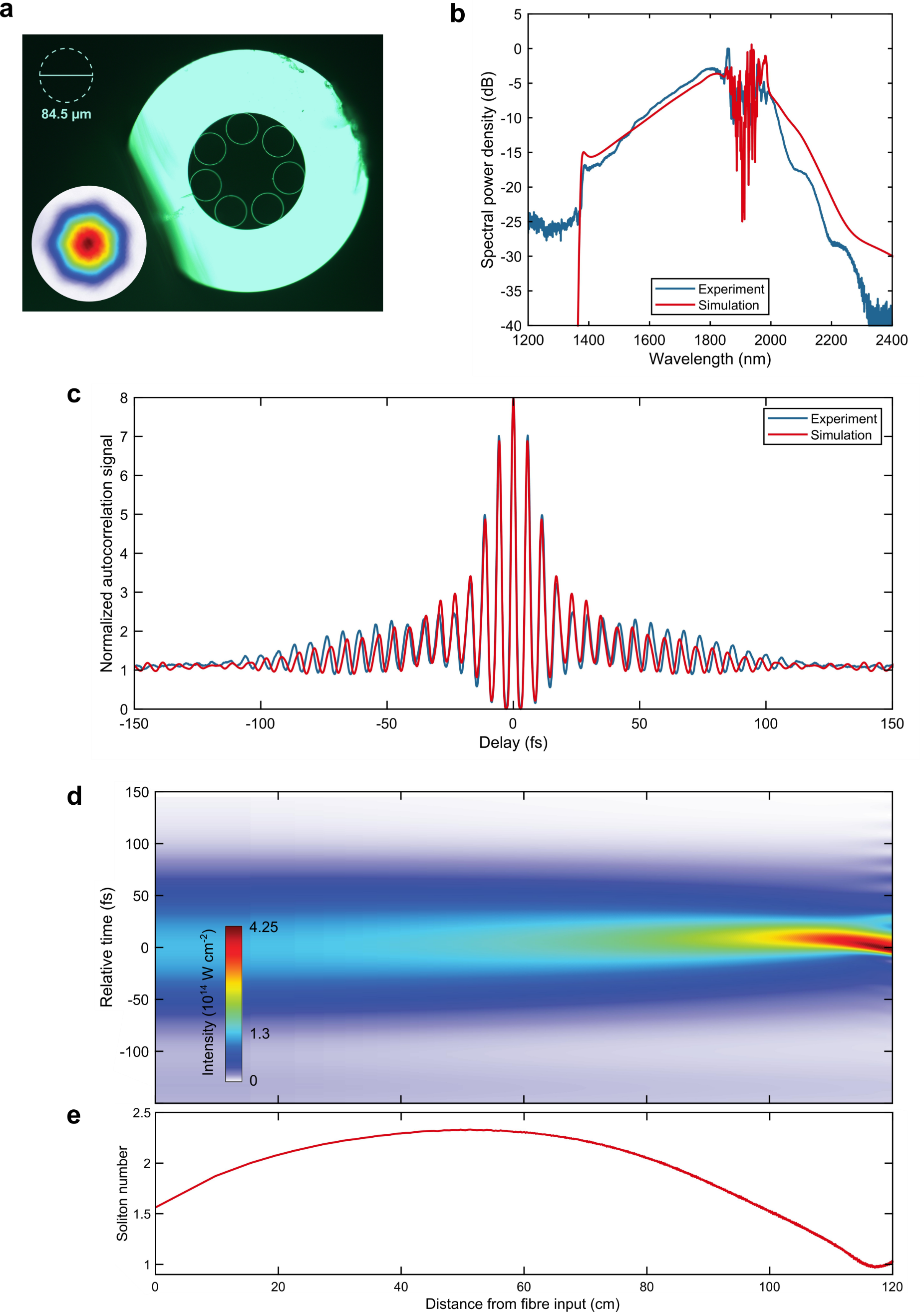


**Fig. S3 Characterization of the self-compressed pulses. a,** Microscopic image of the ARHCF cross-section with a circular scale of the core area and an image of the output beam profile in the top left and bottom left insets, respectively. **b,** Measured and simulated spectra at the ARHCF output for the experimental conditions of optimized HHG. **c,** Measured and simulated autocorrelation traces at the ARHCF output for the experimental conditions of optimized HHG. **d,** Simulated evolution of the time-dependent on-axis intensity. **e,** Calculated soliton number.

**HHG cut-off and signal growth**

To describe the HHG in more detail, the electric field strength, the ionization fraction and the plasma/gas density are used to calculate the coherence length ($L_{\mathrm{coh}}=\pi/\Delta k$, where $\Delta k$ is the wavevector missmatch between the 1650 nm driving field oscillation and the targent wavelength of 4.13 nm (300 eV)) at a position z = 119.65 cm. At this point, the calculated phase velocities, as shown in Fig. 2f of the main text, are equal if we evaluate them directly after the global peak of the electric field. As can be seen in Fig. S4a, that allows the coherence length to be much longer than the absorption length for the duration of the phase-matched half-cycle. Using the semiclassical analysis of HHG^49^ and targeting a short-trajectory photon energy of 300 eV, the relative ionization time within this half-cycle is ~0.4 fs. Note that the relative time axis is centred at the global peak of the electric field and the recombination occurs at ~3.4 fs. The full temporal distribution of HHG photon energy upon recombination vs. relative ionization time is shown in Fig. S4b. In fact, the maximum HHG photon energy for the evaluated half-cycle is 334 eV (ionization time ~0.27 fs, recombination time ~3.8 fs), which is very close to the experimentally observed phase matching cut-off. It was also observed experimentally that the cut-off can be pushed to ~370 eV at the cost of overall yield, when using slightly higher input pulse energy and slightly lower pressure. This observation suggests that the phase matching is tuned to relative ionization times between -2.9 fs and -2.2 fs. Note that the spectroscopy measurements presented in Fig. S6 are performed with a cut-off around 370 eV.


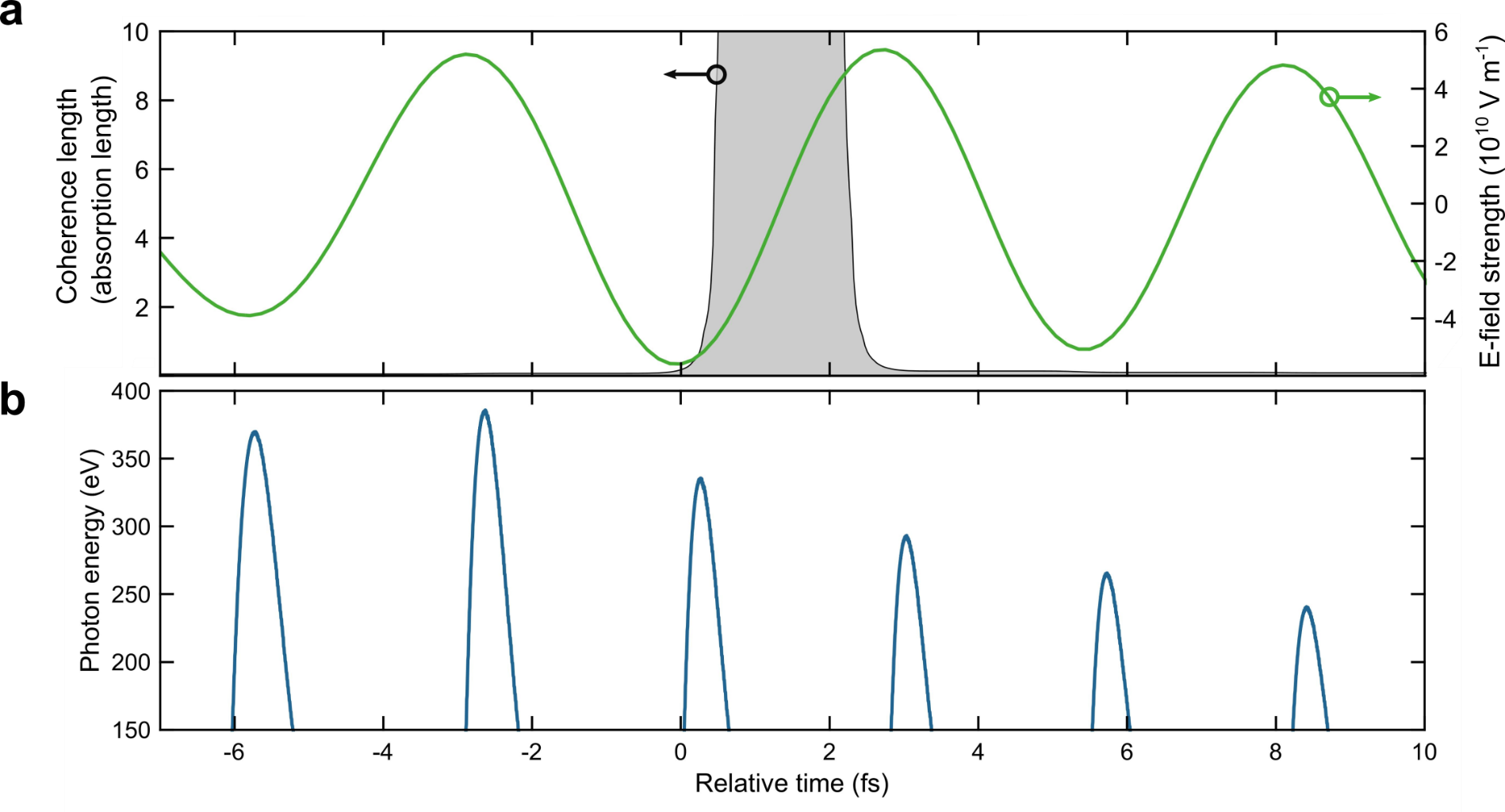


**Fig. S4 HHG phase-matching and photon energy. a,** Electric field strength and phase-matching window at z = 119.65 cm. **b,** Calculated HHG photon energy vs. relative ionization time at z = 119.65 cm.

Fig. 2c and 2d in the main text show that the simulated HHG-relevant ionization fraction is around 3×10^-4^. We note that this ionization level results in the correct amount of spectral blue-shifting when comparing pulse propagation simulations to the experimentally measured output spectra after the ARHCF (see Fig. S3b). However, the critical ionization fraction, at which phase matching is no longer possible, can be calculated to 1×10^-3^. Furthermore, the modal averaging (a result of driving field’s waveguiding) allows to actually exceed this critical fraction by as much as a factor of five^50^. We conclude that nonlinear self-compression to a fundamental, blue-shifting soliton of higher intensity than realized in this first demonstration should still allow for full phase matching, resulting in a dramatically increased dipole moment of the individual HHG emitters and a higher photon energy cut-off. The design of such an optimized pulse self-compression is the subject of research, that is opened up by this demonstration.

As a final step of our HHG analysis, we use the position-dependent pressure p, absorption length L_abs_, 300 eV spectral amplitude of the atomic response A*_q_* and relative phase $\phi$ of the harmonic radiation to estimate its cumulative growth. At this point, it is worth noting that the intrinsic phase of the recombining electrons has been included in $\phi$ as described in^51^. The intrinsic phase was retrieved from the semiclassical analysis of HHG (i.e. the semiclassical action) and checked against the calculations of the semiclassical action in^14^. The number of photons emitted on axis, $N_{\mathrm{ph}}$, as a function of position z is given as^52^:

$$N_{\mathrm{ph}}(z) \sim\left| \int_{0}^{z} {pA_{q}e^{i\phi}\cdot e}^{\left( -\frac{z-z^{'}}{2L_{\mathrm{abs}}} \right)} dz^{'} \right|^{2}$$

where all quantities of the integrant, except for z, have a dependency in the spatial coordinate z’ and are also changing with relative time. The integration along the time of recombination for the short trajectory 300 eV signal gives the result shown in Fig. S5. Starting from 6 mm before the fibre end, we identify an increasing growth of the signal, which peaks close to the position z = 119.65 cm (where $\Delta k$ is exactly 0). After that, the coherence is lost - mostly due to the change in ionization fraction, that is a result of the walk-off between the driving field oscillation and its envelope (see Fig. 2c and 2d of the main text). However, the waveguiding of the driving field allows for a signal growth over almost 3 mm, which is longer than the Rayleigh length at this point (1.8 mm). Clearly, a pulse compression that is tailored to result in electric field waveforms with less sensitivity to temporal walk-off would allow to fully exploit the advantages of the waveguide geometry as our experiments are far from absorption limited (L_abs_ = 7 mm at the fibre end). This would allow for a less significant impact of the walk-off effects on the formal phase matching and on the intrinsic phase of the electrons^14^, resulting in a longer interaction length and a significantly increased photon flux at the fibre output.


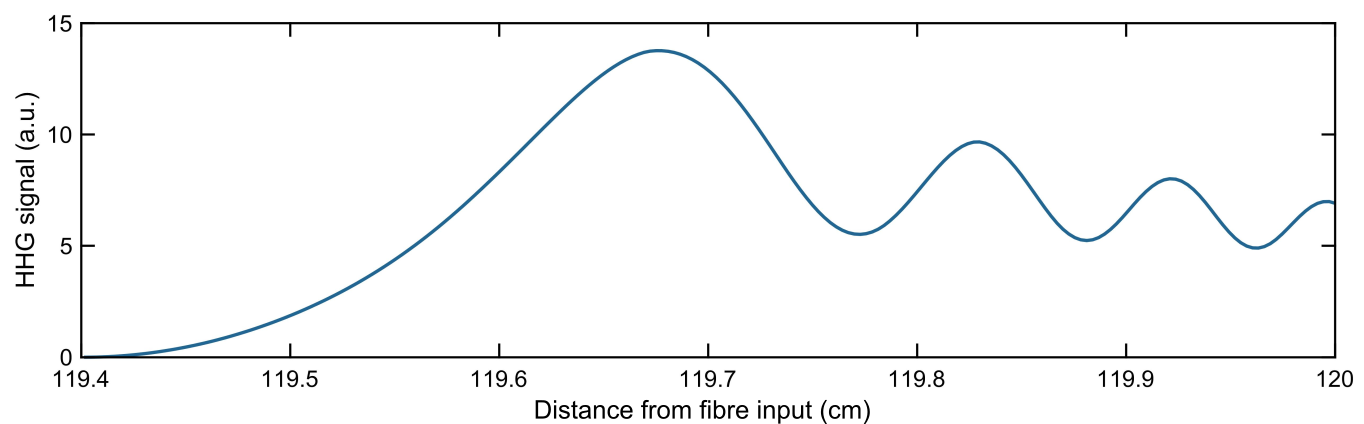


**Fig. S5 Calculated growth of HHG signal.** The blue line represents the growth of the HHG signal at 300 eV, considering the short trajectories and the phase-matched strongest electric field half-cycle only.

**Near-edge X-ray absorption fine structure spectroscopy**

To show the applicability of our source, we demonstrate a near-edge X-ray absorption fine structure spectroscopy measurement using a 500 nm thick mylar filter. The reduced chemical structure of the fundamental part of the polyethylene terephthalate molecule can be seen in the inset of Fig. S6. At photon energies just below the carbon K-edge as well as at >320 eV, the measured optical density (OD) is very close to the theoretical expectation for a 500 nm thick C_10_H_8_O_4_ film, without considering the molecular bonding environment. The optical density is calculated as:

$OD=-log\left( \frac{S_{\mathrm{Filter}}}{S_{\mathrm{In}}} \right)$

where *S*_Filter_ and *S*_In_ are the spectral power densities with and without the mylar filter in the beam path. Close to the carbon K-edge, it is possible to observe the signature of the molecular bonding environment. The sharp peak around 285 eV is due to transitions to the antibonding *π** orbital of C=C within the aromatic ring structure^53,54^. Similarly, the relatively broad hump between 295 eV and 310 eV can be associated with the antibonding *σ** orbital^53,55,56^. Absorption features that explain the structure around 289 eV can be found in the transition to the antibonding *π** orbital of C=O^56,57^.


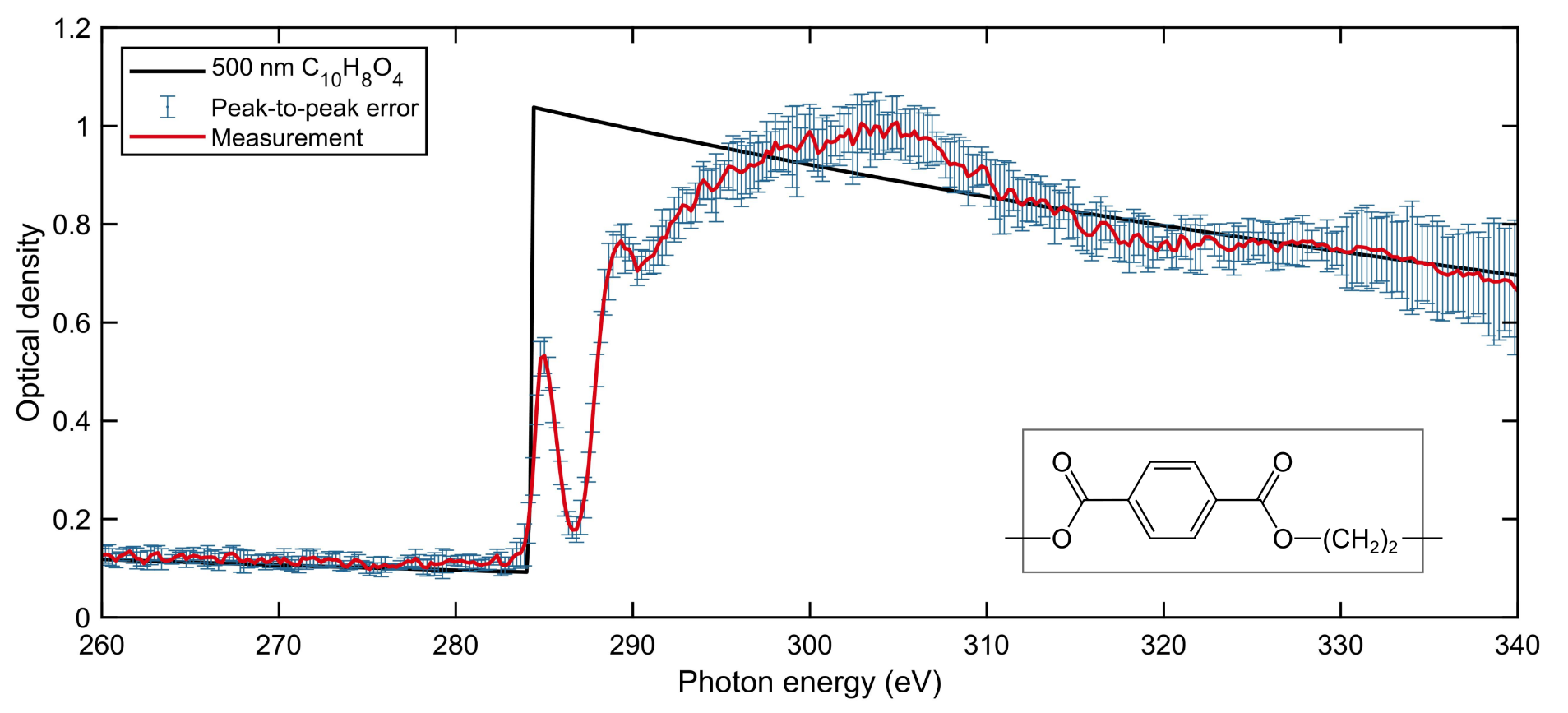


**Fig. S6 NEXAFS characterization of a 500 nm thick mylar foil.** The figure shows the experimental NEXAFS optical density, which was extracted from two consecutive measurements (with and without transmission through the mylar foil) at 30 seconds integration time. While the red line represents data corresponding to a single OD measurement without averaging, the blue error bars show the peak-to-peak deviations of four consecutively recorded NEXAFS spectra. The measurement is compared to the absorption edge of the weighted chemical compounds of mylar, which simplified molecular “unit cell” is depicted in the inset. The OD data that neglect the molecular bonding environment (black line) have been retrieved from ref.^34^.

**Long-term stability measurement of the power in the water window**

The overall optical power in the water window is estimated to >1 nW with an RMS deviation of about 5.5% over 20 minutes, when the sampling interval is set to 5 seconds. The duration of the characterization time shown in Fig. S7 corresponds to about 120 million laser shots, supporting the suitability of the concept demonstrated herein for applications that require high repetition rates in the SXR^15–17^. Additionally, it can be seen from the data presented in Fig. S7 that most of the fluctuations manifest themselves on a timescale related to thermal/mechanical drifts. Consequently, the stability of this proof-of-principal SXR source can be improved significantly by adding a slow feedback loop to the experimental conditions like the laser power and/or gas pressure applied to the high-pressure chamber.


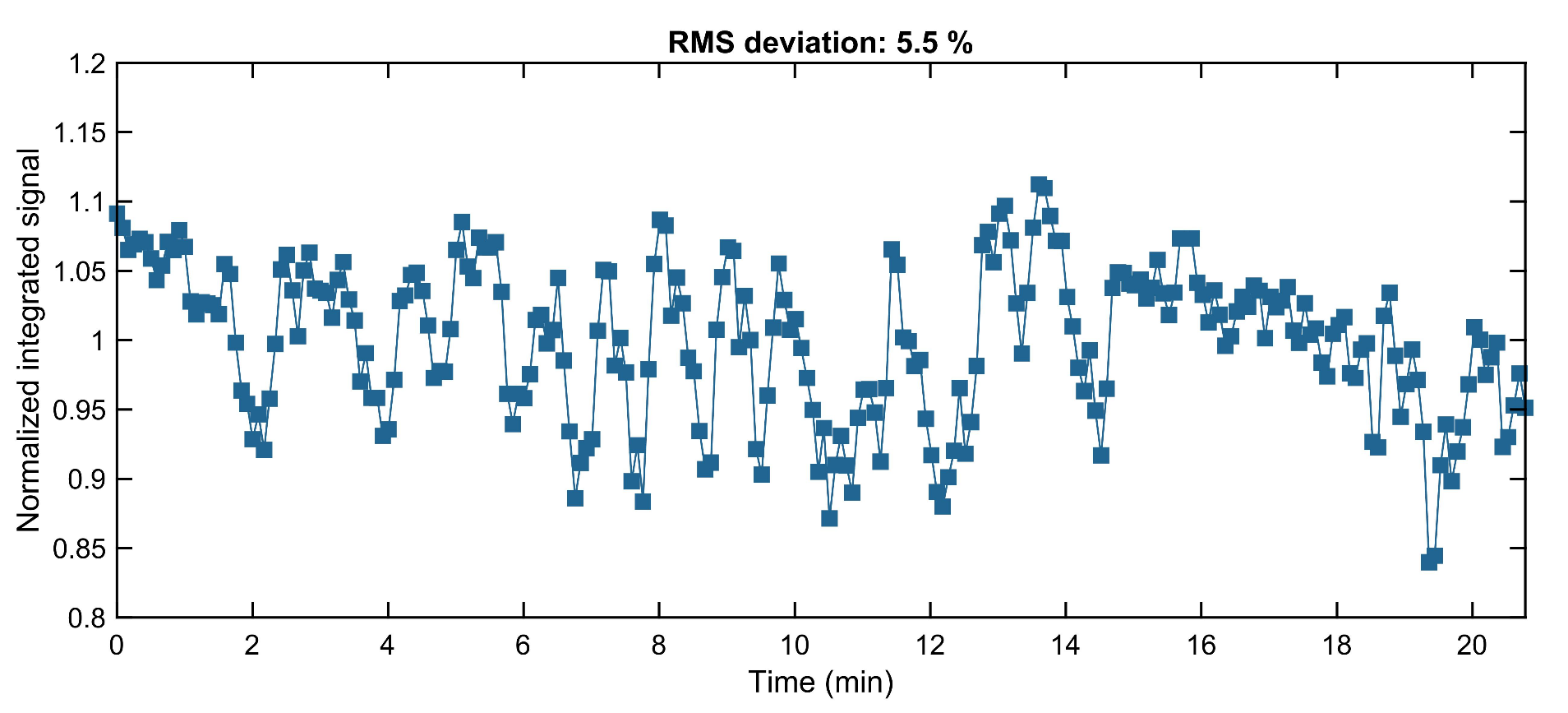


**Fig. S7 Long term characterization of the integrated power in the water window.** Integrated signal at photon energies between 284 eV and 340 eV, measured over a duration of about 20 minutes with an individual integration time of 5 seconds. The measured data points are represented by the solid blue squares, connected by the thin blue line to guide the eye.

**References**

1. Rundquist, A. *et al.* Phase-Matched Generation of Coherent Soft X-rays. *Science* **280**, 1412–1415 (1998).

2. Young, L. *et al.* Roadmap of ultrafast x-ray atomic and molecular physics. *Journal of Physics B*: *Atomic*, *Molecular and Optical Physics* **51**, 032003 (2018).

3. Pertot, Y. *et al.* Time-resolved x-ray absorption spectroscopy with a water window high-harmonic source. *Science* ***355***, 264–267 (2017).

4. Popmintchev, D. *et al.* Near- and Extended-Edge X-Ray-Absorption Fine-Structure Spectroscopy Using Ultrafast Coherent High-Order Harmonic Supercontinua. *Physical Review Letters* **120**, 093002 (2018).

5. Rose, M. *et al.* Quantitative ptychographic bio-imaging in the water window. *Optics* *Express* **26**, 1237-1254 (2018).

6. Gaumnitz, T. *et al.* Streaking of 43-attosecond soft-X-ray pulses generated by a passively CEP-stable mid-infrared driver. *Optics* *Express* **25**, 27506-27518 (2017).

7. Li, J. *et al.* 53-attosecond X-ray pulses reach the carbon K-edge. *Nature* *Communications* **8**, 186 (2017).

8. Stein, G. J. *et al*. Water-window soft x-ray high-harmonic generation up to the nitrogen K-edge driven by a kHz, 2.1 *μ*m OPCPA source. *Journal of Physics B*: *Atomic*, *Molecular and Optical Physics* **49**, 155601 (2016).

9. Feng, T. *et al.* 27 W 21 µm OPCPA system for coherent soft X-ray generation operating at 10 kHz. *Optics* *Express* **28**, 8724-8733 (2020).

10. Johnson, A. S. *et al.* High-flux soft x-ray harmonic generation from ionization-shaped few-cycle laser pulses. *Science Advances* **4**, eaar3761 (2018).

11. Cousin, S. L. *et al.* High-flux table-top soft x-ray source driven by sub-2-cycle, CEP stable, 185-μm 1-kHz pulses for carbon K-edge spectroscopy. *Optics* *Letters* **39**, 5383-5386 (2014).

12. Chen, M.-C. *et al.* Bright, Coherent, Ultrafast Soft X-Ray Harmonics Spanning the Water Window from a Tabletop Light Source. *Physical Review Letters* **105**, 173901 (2010).

13. Shiner, A. D. Wavelength Scaling of High Harmonic Generation Efficiency. *Physical Review Letters* **103**, 073902 (2009).

14. Hernández-García, C. *et al.* Group velocity matching in high-order harmonic generation driven by mid-infrared lasers. *New Journal of Physics* **18**, 073031 (2016).

15. Pupeikis, J. *et al.* Water window soft x-ray source enabled by a 25 W few-cycle 22 µm OPCPA at 100 kHz. *Optica* **7**, 168-171 (2020).

16. Rothhardt, J. *et al.* High-repetition-rate and high-photon-flux 70 eV high-harmonic source for coincidence ion imaging of gas-phase molecules. *Optics* *Express* **24**, 18133-18147 (2016).

17. Chiang, C.-T. *et al.* Boosting laboratory photoelectron spectroscopy by megahertz high-order harmonics. *New Journal of Physics* **17**, 013035 (2015).

18. Travers, J. C., Grigorova, T. F., Brahms, C. & Belli, F. High-energy pulse self-compression and ultraviolet generation through soliton dynamics in hollow capillary fibres. *Nature* *Photonics* **13**, 547-554 (2019).

19. Schnürer, M. *et al.* Guiding and high-harmonic generation of sub-10-fs pulses in hollow-core fibers at 1015 W/cm2. *Applied* *Physics* *B* **67**, 263-266 (1998).

20. Travers, J. C. *et al*. Ultrafast nonlinear optics in gas-filled hollow-core photonic crystal fibers [Invited]. *Journal of the Optical Society of America B* **28**, A11-A26 (2011).

21. Yu, F. & Knight, J. C. Negative Curvature Hollow-Core Optical Fiber. *IEEE Journal of Selected Topics in Quantum Electronics* **22**, 4400610 (2016).

22. Wiegandt, F. *et al.* Quasi-phase-matched high-harmonic generation in gas-filled hollow-core photonic crystal fiber. *Optica* **6**, 442-447 (2019).

23. Gebhardt, M. *et al.* Nonlinear pulse compression to 43  W GW-class few-cycle pulses at 2  μm wavelength. *Optics* *Letters* **42**, 4179-4182 (2017).

24. Saleh, M. F. *et al.* Theory of Photoionization-Induced Blueshift of Ultrashort Solitons in Gas-Filled Hollow-Core Photonic Crystal Fibers. *Physical Review Letters* **107**, 203902 (2011).

25. Kazamias, S. *et al.* Pressure-induced phase matching in high-order harmonic generation. *Physical Review A* **83**, 063405 (2011).

26. Elu, U. *et al.* High average power and single-cycle pulses from a mid-IR optical parametric chirped pulse amplifier. *Optica* **4**, 1024-1029 (2017).

27. Jin, C., Wang, G., Wei, H., Le, A.-T. & Lin, C. D. Waveforms for optimal sub-keV high-order harmonics with synthesized two- or three-colour laser fields. *Nature* *Communications* **5**, 4003 (2014).

28. Michieletto, M. *et al.* Hollow-core fibers for high power pulse delivery. *Optics* *Express* **24**, 7103-7119 (2016).

29. Tani, F. *et al*. Effect of anti-crossings with cladding resonances on ultrafast nonlinear dynamics in gas-filled photonic crystal fibers. *Photonics* *Research* **6**, 84-88 (2018).

30. Livesey, R. G. Solution methods for gas flow in ducts through the whole pressure regime. *Vacuum* **76**, 101–107 (2004).

31. Henningsen, J. & Hald, J. Dynamics of gas flow in hollow core photonic bandgap fibers. *Applied* *Optics* **47**, 2790-2797 (2008).

32. Tani, F., Travers, J. C. & St.J. Russell, P. Multimode ultrafast nonlinear optics in optical waveguides: numerical modeling and experiments in kagomé photonic-crystal fiber. *Journal of the Optical Society of America* *B* **31**, 311-320 (2014).

33. Ammosov, M. V., Delone, N. B. & KraǏnov, V. P. Tunnel ionization of complex atoms and of atomic ions in an alternating electromagnetic field. *Soviet Physics -* *Journal of Experimental and Theoretical Physics* **64**, 1191-1194 (1986).

34. Henke, B. L., Gullikson, E. M. & Davis, J. C. X-Ray Interactions: Photoabsorption, Scattering, Transmission, and Reflection at E = 50-30,000 eV, Z = 1-92. *Atomic Data and Nuclear Data Tables* **54**, 181-342 (1993).

35. Hädrich, S. *et al.* High photon flux table-top coherent extreme-ultraviolet source. *Nature* *Photonics* **8**, 779-783 (2014).

36. Frassetto, F. *et al.* Compact spectrometer for the analysis of high harmonics content of extreme-ultraviolet free-electron-laser radiation. Proceedings of SPIE 7802, Advances in X-Ray/EUV Optics and Components V. San Diego, California, USA: SPIE, 2010, 780209.

37. Ding, C. *et al.* High flux coherent super-continuum soft X-ray source driven by a single-stage, 10mJ, Ti:sapphire amplifier-pumped OPA. *Optics Express* **22**, 6194-6202 (2014).

38. Rothhardt, J. *et al*. 53 W average power few-cycle fiber laser system generating soft x rays up to the water window. *Optics Letters* **39**, 5224–5227 (2014).

39. Schmidt, C. *et al.* High-order harmonic source spanning up to the oxygen K-edge based on filamentation pulse compression. *Optics Express* **26**, 11834-11842 (2018).

40. Cardin, V. *et al.* Self-channelled high harmonic generation of water window soft x-rays. *Journal of Physics B*: *Atomic*, *Molecular and Optical Physics* **51**, 174004 (2018).

41. Fu, Y. *et al.* High efficiency ultrafast water-window harmonic generation for single-shot soft X-ray spectroscopy. *Communications Physics* **3**, 92 (2020).

42. Barreau, L. *et al.* Efficient table-top dual-wavelength beamline for ultrafast transient absorption spectroscopy in the soft X-ray region. *Scientific Reports* **10**, 5773 (2020).

43. Spielmann, C. Generation of Coherent X-rays in the Water Window Using 5-Femtosecond Laser Pulses. *Science* **278**, 661–664 (1997).

44. Gibson, E. A. Coherent Soft X-ray Generation in the Water Window with Quasi-Phase Matching. *Science* **302**, 95–98 (2003).

45. Takahashi, E. J. *et al*. Coherent Water Window X Ray by Phase-Matched High-Order Harmonic Generation in Neutral Media. *Physical Review Letters* **101**, 253901 (2008).

46. Limpert, J. *et al.* Yb-doped large-pitch fibres: effective single-mode operation based on higher-order mode delocalisation. *Light: Science & Applications* **1**, e8 (2012).

47. Agrawal, G. *Nonlinear Fiber Optics*. (Academic Press, 2001).

48. Chang, W., Hölzer, P., Travers, J. C. & Russell, P. S. J. Combined soliton pulse compression and plasma-related frequency upconversion in gas-filled photonic crystal fiber. *Optics Letters* **38**, 2984-2987 (2013).

49. Lewenstein, M. *et al.* Theory of high-harmonic generation by low-frequency laser fields. *Physical Review A* **49**, 2117–2132 (1994).

50. Durfee, C. G. *et al.* Phase Matching of High-Order Harmonics in Hollow Waveguides. *Physical Review Letters* **83**, 2187–2190 (1999).

51. Popmintchev, T. *et al.* The attosecond nonlinear optics of bright coherent X-ray generation. *Nature Photonics* **4**, 822–832 (2010).

52. Constant, E. *et al.* Optimizing High Harmonic Generation in Absorbing Gases: Model and Experiment. *Physical Review Letters* **82**, 1668–1671 (1999).

53. Stöhr, J. *NEXAFS Spectroscopy*. (Springer-Verlag, 1992).

54. Sham, T.-K. *Chemical Applications of Synchrotron Radiation*. (World Scientific, 2002).

55. Rocco, M. L. M. *et al.* High-Resolution Near-Edge X-ray Absorption Fine Structure Study of Condensed Polyacenes. *Journal of Physical Chemistry C* **122**, 28692–28701 (2018).

56. Okajima, T. *et al.* Polarized NEXAFS Spectroscopic Studies of Poly(butylene terephthalate), Poly(ethylene terephthalate), and Their Model Compounds. *Journal of Physical Chemistry A* **102**, 7093–7099 (1998).

57. Dhez, O., Ade, H. & Urquhart, S. Calibrated NEXAFS spectra of some common polymers. *Journal of Electron Spectroscopy and Related Phenomena* **128**, 85–96 (2003).
